# Frontline gefitinib in advanced non-small cell lung cancer: Meta-analysis of published randomized trials

**DOI:** 10.4103/1817-1737.65047

**Published:** 2010

**Authors:** Ezzeldin M. Ibrahim

**Affiliations:** Oncology Center of Excellence, International Medical Center, Jeddah, Saudi Arabia

**Keywords:** EGFR, gefitinib, non-small cell lung cancer

## Abstract

**OBJECTIVE:**

Gefitinib, a small molecule tyrosine kinase inhibitor, showed a substantial effect as a salvage treatment for patients with advanced non-small cell lung cancer (NSCLC) who had failed prior chemotherapy. Subsequent phase III trials in previously untreated patients have failed to demonstrate such benefit. It was later reported that gefitinib had a positive outcome when used in selected population.

**RATIONAL:**

The inconsistent results and the lack published meta-analysis that systematically examined the overall efficacy of gefitinib in the frontline setting in such patients, have prompted the current meta-analysis.

**METHODS:**

We selected for analysis only those randomized, peer-reviewed clinical studies where the efficacy of gefitinib-based therapy (GBT) was investigated in chemotherapy naïve patients with locally advanced or metastatic NSCLC. We also included studies where patients were randomized between gefitinib vs. placebo or none after initial chemoradiation or chemotherapy induction offered to all included patients.

**RESULTS:**

We identified seven eligible studies involving 2,646 and 1,939 patients randomized to GBT and to control arms, respectively. In mostly unselected population, GBT was not associated with higher objective response rate (ORR), progression-free survival (PFS) (hazard ratio [HR] = 0.97, 95% CI: 0.78–1.20, *P* = 0.78), or overall survival (OS) (HR = 1.04, 95% CI: 0.95–1.13, *P* = 0.45) as compared with control interventions. In a fraction of patients with known EGFR mutation status, GBT showed significantly higher ORR among patients with mutant EGFR (odds ratio [OR] = 2.81, 95% CI: 1.71–4.62, *P* < 0.0001); however, EGFR mutation was not associated with better PFS or OS with GBT. Nevertheless, patients receiving GBT experienced significant improvement in quality of life as compared with those in the control arms.

**CONCLUSION:**

We conclude that GBT cannot be recommended for frontline management of patients with advanced NSCLC in unselected patient population.

Lung cancer remains the highest cause of cancer-related mortality. In patients with locally advanced and metastatic non-small cell lung cancer (NSCLC), short-lived responses to aggressive chemotherapy are observed in approximately 30% of patients, the impact on patient survival has been modest.[1]

The small molecule tyrosine kinase inhibitor (TKI), gefitinib (Iressa; AstraZeneca, Wilmington, DE), targets the epidermal growth factor receptor (EGFR) was tested in chemotherapy-refractory NSCLC patients, on the basis of their frequent expression of EGFR and their poor response to standard therapies. In two large phase II trials (the Iressa Dose Evaluation in Advanced Lung Cancer [IDEAL] 1 study and the IDEAL 2 study), the results indicated that gefitinib had a substantial effect as a salvage treatment for patients who had failed at least one or two previous regimens of chemotherapy.[2,3]

Two subsequent phase III trials randomized previously untreated patients with advanced NSCLC to standard platinum-based chemotherapy, with or without the addition of gefitinib at two doses.[4,5] These trials reported no difference in objective response rate (ORR), progression-free survival (PFS), or overall survival (OS) with the addition of gefitinib to standard chemotherapy.

While initial trials of gefitinib failed to show activity in most cases of NSCLC, a subset of cases that did respond had rapid and dramatic tumor shrinkage. These responses were more common in women, East Asians, and nonsmokers, and their tumors were primarily adenocarcinomas. It was later reported that the majority of tumors with dramatic responses harbor mutations in the EGFR kinase domain that were not found in nonresponsive cases.[6–8] Moreover, other phase III studies have reported positive outcome when gefitinib was used in selected population.[9]

The inconsistent results, the intriguing role of EGFR mutations, the influence of patients selection, and the lack published meta-analysis that systematically examined the overall efficacy of gefitinib in the frontline setting in patients with locally advanced or metastatic NSCLC, have prompted the current meta-analysis that intended to examine the potential benefit of gefitinib in that setting.

## Methods

### Literature search

We did a comprehensive search of citations from PubMed, proceedings of the main oncology conferences, Cochrane Central Register of Controlled Trials, Cochrane Database of Systematic Reviews, and Database of Abstracts of Review of Effectiveness. The search was limited to randomized, peer-reviewed clinical studies and reviews in English language. Our initial search through each resource used queries with the medical subject headings (MeSH) terms: “lung neoplasm”, OR “lung cancer” AND “gefitinib”. The search strategy also used several text terms to identify relevant information. Reference lists from relevant primary studies and review articles were examined to find other additional publications.

### Study selection

We selected for analysis only those randomized, peer-reviewed clinical studies where the efficacy of gefitinib-based therapy (GBT) was investigated in chemotherapy naïve patients with locally advanced or metastatic NSCLC. We also included studies where patients were randomized between gefitinib vs. placebo or none after initial chemoradiation or chemotherapy induction offered to all included patients.

### Statistical methods

Before performing the analyses, data of each study were carefully checked and verified for coherence with the original publications. Data were entered in a computer database for transfer and statistical analysis in Review Manager Version 5.0.17 (Cochrane Collaboration, Software Update, Oxford, UK) and Comprehensive Meta Analysis Version 2.2.048 (NJ, USA). For trials included in this meta-analysis, if log hazard ratio (HR) and its variance were not presented explicitly, appropriate estimations methods were used to extract estimates of these statistics.[10,11]

In this meta-analysis, both fixed and random effect models were tested where appropriate.[12,13] *X*^2^ tests were used to study heterogeneity between trials. *I*^2^ statistic was used to estimate the percentage of total variation across studies, due to heterogeneity rather than chance. If the *P* value was ≤0.1, the assumption of homogeneity was deemed invalid, and the random-effects model was reported after exploring the causes of heterogeneity.[14] A two-tailed *P* value of <0.05 was considered statistically significant. Publication bias was explored through visual inspection of the funnel plots.[13] Findings of the meta-analysis are depicted in classical Forest plots, with point estimates and 95% confidence interval (CI) for each trial and overall; size of the squares is proportional to effect size.

## Results

### Studies and patient characteristics

After exclusion of duplicate and irrelevant studies, our search yielded seven eligible published studies that were retrieved for evaluation that is more detailed. There were 2,646 and 1,939 patients randomized to GBT and to the control arms, respectively. Of the included studies, four studies compared gefitinib plus chemotherapy vs. chemotherapy alone,[5,15–17] two studies compared gefitinib alone vs. chemotherapy,[9,18] and one study compared gefitinib plus best supportive care (BSC) vs. BSC alone.[19] Analysis of the efficacy of gefitinib in the Iressa NSCLC Trial Assessing Combination Treatment (INTACT-1)[15] and INTACT-2[5] studies based on EGFR expression, mutations, and gene amplification were also included.[6] Table 1 depicts the main characteristics of the included studies, whereas Table 2 shows the summary of the efficacy data.

**Table 1:** Characteristics of the seven studies included in the meta-analysis

Study	Description and patient selection	Gefitinib-based therapy/control														
		No.	Median age	Male %	Stage %		Histology	Performance status			Race				Smoking	
					IIIB	IV	AC%	PS 0	PS 1	PS 2	White	Asian	Black	Other	Ever	Never
Giaccone 2004[15]	Phase III randomized, double-blind, placebo-controlled (INTACT-1): Gemcitabine + cisplatin + gefitinib 500 mg vs. gemcitabine + cisplatin + gefitinib 250 mg vs. gemcitabine + cisplatin + placebo Unselected patients.	365/365/363	61/59/61	72/77/72	30/26/28	67/72/69	43/49/47	32/34/34	58/56/56	10/10/10	91/90/90	2/2/1	8/4/5	8/4/5	NR 32/32	NR 32/32
Herbst *et al*. 2004[5]	Phase III randomized, double-blind, placebo-controlled (INTACT-2). Paclitaxel + carboplatin + gefitinib 500 mg vs. paclitaxel + carboplatin + gefitinib 250 mg vs. paclitaxel + carboplatin + placebo Unselected patients.	347/345	62/61/63	40/42/39	15/16/17	82/81/78	58/56/52	35/33/39	52/57/52	13/10/9	89/90/92		8/4/5	4/6/3	NR	NR
Kelly *et al*. 2008[17]	Phase III randomized, placebo-controlled. Concurrent radiotherapy and etoposide- cisplatin followed by docetaxel. then randomization to gefitinib vs. placebo. Patients with Stage III and PS 0 or 1, no plural or pericardial effusion.	118/125	62/61	79/74	IIIA 45/51 IIIB 55/49		30/33	51/56	45/40		97/86	0/2	3/11	0/2	NR	NR
Takeda *et al*. 2010[16]	Phase III randomized, open-label. Platinum-doublet chemotherapy followed by gefitinib vs. platinum-doublet chemotherapy followed by platinum-doublet chemotherapy. All Japanese patients.	300/298	62/63	64/64	18/18	82/82	79/78	30/35	70/65			100/100			67/70	32/30
Crino *et al*. 2008[18]	Phase II, randomized, open-label (INVITE). Gefitinib vs. vinorelbine. Patients ≥ 70 years of age.	97/99	74/74	77/74	20/26	80/74	35/45	13/21	63/62	24/16	81/84	18/14		1/2	82/89	18/11
Mok *et al*. 2009[9]	Phase III randomized, open-label. Gefitinib vs. paclitaxel- carboplatin. Patients non- or former light-smoker, East Asian, with AC.	609/	57/57	21/21	25/24	75/76	100/100	26/26	64/63	10/11		100/100			6/6	94/94
Goss *et al*. 2009[19]	Phase II randomized, placebo-controlled. Gefitinib-BSC vs. BSC only. Patients with WHO PS 2-3.	100/101	74/76	61/60	16/17	84/83	45/46			PS 2 55/62 PS3 45/38	96/96	4/3		0/1	90/91	10/9

AC, Adenocarcinoma; PS, performance status; NR, not reported.

**Table 2:** Summary of the efficacy analysis of the seven studies included in the meta-analysis

	Primary end point	Median FU (ms)	ORR%			PFS (months)			OS (months)		
			GBT	Control	*P*	GBT	Control	*P*	GBT	Control	*P*
Giaccone 2004[15]	OS	15.9	50*	47	NS	5.5*	6.0	NS	9.9*	10.9	NS
Herbst *et al*. 2004[5]	OS	6.0 (PFS)	30*	29	NS	4.6*	5.0	NS	8.7*	9.9	NS
		12.0 (OS)	30+		NS	5.3+			9.8*		
Kelly *et al*. 2008[17]	OS	27.0 (pre-mature closure)	Not applicable			8.3	11.7	NS	23.0	35.0	0.013
Takeda *et al*. 2010[16]	OS	NR	34	29	NS	4.6	4.3	<0.001	13.7	12.9	NS
Crino *et al*. 2008[18]	PFS	6.0	3	5	NS	2.7	2.9	NS	5.9	8.0	NS
Mok *et al*. 2009[9]	PFS	5.6	43	32	<0.001	5.7	5.8	<0.001	18.7	17.3	NS
Goss *et al*. 2009[19]	PFS	1.3 (PFS)	6	1	NS	1.4	1.4	NS	3.7	2.8	NS
		~3.0 (OS)									

NS, Not significant; OS, overall survival; PFS, progression-free survival

* Gefitinib 500 mg

+ gefitinib 250 mg

### Objective response rate

**Figure 1:**
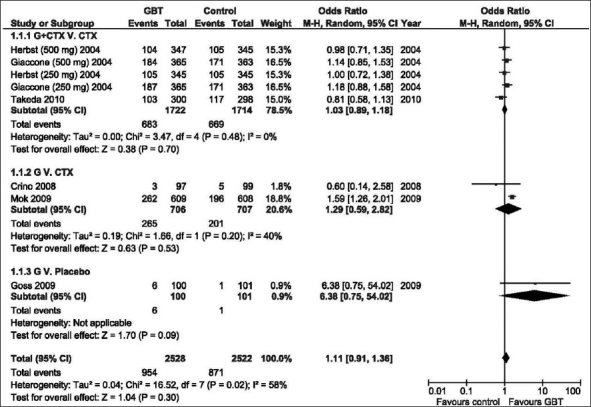
Odds ratio of objective response rate of GBT vs. control interventions (random effects model). G, Gefitinib

**Figure 2:**
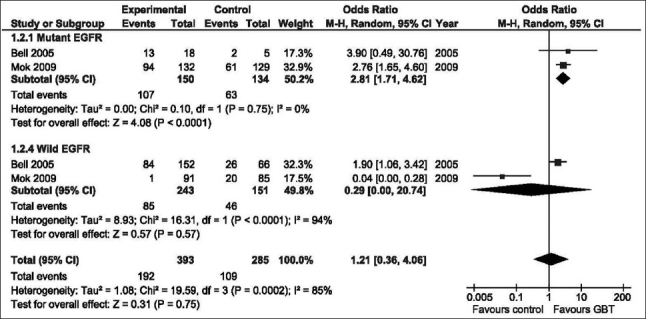
Odds ratio of objective response rate of GBT vs. control interventions according to EGFR mutation status (random effects model). G, Gefitinib

There was significant heterogeneity between studies (*P* = 0.02); therefore, the random effects model was examined. Figure 1 shows that in none of the comparisons was the ORR significantly different in the GBT vs. the control interventions. Of the original INTACT-1[15] and INTACT-2[5] trials, molecular analysis for EGFR mutation status was known in 150 patients (7%),[6] whereas the status was known in 437 patients (36%) of the study of Mok *et al*.[9] Figure 2 shows that GBT was associated with almost threefold higher ORR compared with the control regimens among patients with positive EGFR mutation (OR = 2.81, 95% CI: 1.71–4.62, *P* < 0.0001). On the other hand, no benefit was demonstrated among mutation-negative patients. The positive effect in mutation-positive patients was mainly attributed to the outcome of the Mok *et al*. trial that included select population [Table 1].[9]

### Progression-free survival

**Figure 3:**
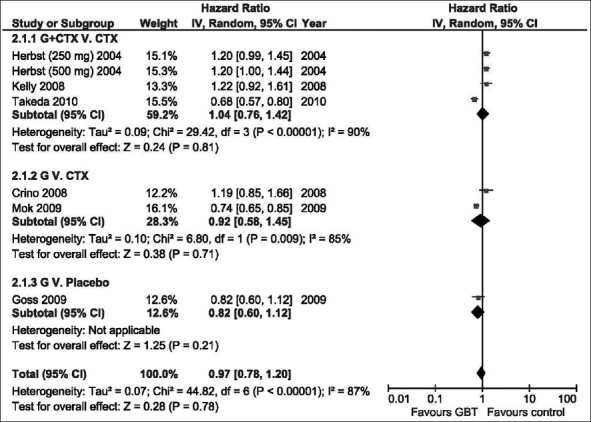
The hazard ratio for progression-free survival of GBT vs. control interventions (random effects model). G; Gefitinib

Analysis of PFS using the random effects model [Figure 3], failed to show any significant benefit of GBT vs. control regardless of trials designs (HR = 0.97, 95% CI: 0.78–1.20, *P* = 0.78), neither was any PFS advantage was found among patients with mutant or wild EGFR [Table 3].

**Table 3:** Efficacy of Gefitinib-based therapy vs. control according to EGFR mutation status and histology

Study	PFS	*P* value	Study	OS	*P* value
	HR (95% CI)			HR (95% CI)	
**EGFR mutant**					
Bell *et al*. 2005[6]	0.55 (0.19–1.60)		Bell *et al*. 2005[6]	1.77 (0.50–6.25)	
Crino *et al*. 2008[18]	3.13 (1.45–6.76)		Crino 2008[18]	2.88 (1.21–6.85)	
Goss *et al*. 2009[19]	0.29 (0.11–0.75)		Goss *et al*. 2009[19]	0.44 (0.17–1.13)	
Mok *et al*. 2009[9]	0.48 (0.36–0.64)		Mok *et al*. 2009[9]	0.78 (0.49–1.23)	
Subtotal	0.71 (0.27–1.85)	0.48		1.10 (0.51–2.40)	0.81
EGFR wild					
Bell *et al*. 2005[6]	0.73 (0.53–1.01)		Bell *et al*. 2005[6]	0.91 (0.76–1.23)	
Goss *et al*. 2009[19]	0.74 (0.38–1.45)		Goss *et al*. 2009[19]	1.02 (0.56–1.87)	
Mok *et al*. 2009[9]	2.85 (2.05–3.97)		Mok *et al*. 2009[9]	1.38 (0.92–2.08)	
Subtotal	1.17 (0.43–3.19)	0.76		1.06 (0.81–1.39)	0.65
Total	0.89 (0.45–1.76)	0.74		1.06 (0.77–1.47)	0.71
**Adenocarcinoma**					
			Herbst *et al*. 2004[5]		
			500 mg	1.03 (0.81–1.31)	
			250 mg	1.16 (0.90–1.48)	
			Takeda *et al*. 2010[16]	0.79 (0.64–0.97)	
Subtotal				0.97 (0.77–1.22)	0.81
**Non-adenocarcinoma**					
			Herbst *et al*. 2004[5]		
			500 mg	0.74 (0.52–1.04)	
			250 mg	0.92 (0.64–1.32)	
			Takeda *et al*. 2010[16]	1.24 (0.85–1.80)	
Subtotal				0.94 (0.70–1.26)	0.67
Total				0.96 (0.81–1.13)	0.60

### Overall survival

**Figure 4:**
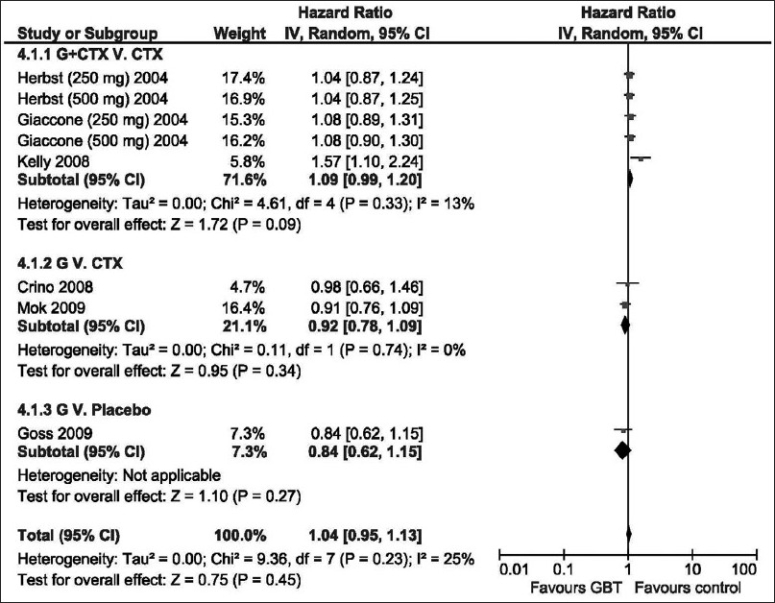
The hazard ratio for overall survival of GBT vs. control interventions (random effects model). G, Gefitinib

Similar to the outcome of PFS analysis, using the random effects model, could not demonstrate significant OS advantage of GBT vs. control in the different trials designs [Figure 4]; HR = 1.04 (95% CI: 0.95–1.13, *P* = 0.45). Statistically significant OS survival for GBT was not demonstrated regardless of tumor histology (adenocarcinoma vs. non-adenocarcinoma), or EGFR mutation status [Table 3].

### Analysis of PFS and OS according to other prognostic features

There were no adequate reported data to allow analysis of PFS according to race, age, gender, or histology. Neither were adequate data for analyzing OS according to race, age, or gender. Combining the data of the studies of Mok *et al*.[9] and Takeda *et al*.[16] that included only Asian population showed that there was a significant PFS benefit from GBT vs. non-gefitinib interventions (HR = 0.72, 95% CI: 0.65–0.08, P < 0.0001); however, the difference in ORR was not different (HR = 1.15, 95% CI: 0.59–2.22, *P* = 0.69). OS analysis was not possible as there were no enough survival data reported from the study of Takeda *et al*.[16]

### Quality of life

**Figure 5:**
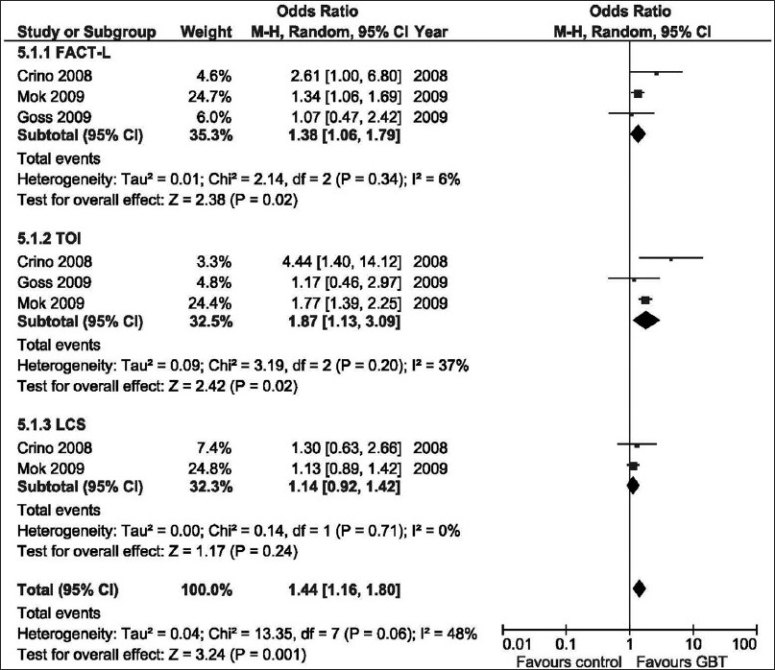
Odds ratio of quality of life assessment of GBT vs. control interventions (random effects model). FACT-L, Functional Assessment of Cancer Therapy-Lung; G, Gefitinib; LCS, Lung–cancer subscale; TOI, Trial outcome index

In three studies,[9,18,19] there were adequate reported data that allowed analysis of the effect of GBT on the QOL. QOL was assessed with the use of the Functional Assessment of Cancer Therapy–Lung (FACT-L) questionnaire, and the Trial Outcome Index (TOI), which is the sum of the physical well-being, functional well-being, and the lung-cancer subscale (LCS) scores of FACT-L. Figure 5 shows that significantly more patients in the GBT than in the control had an improvement in QOL as assessed by scores on the FACT-L questionnaire (OR = 1.38; 95% CI: 1.06–1.79; *P* = 0.02) and by scores on the TOI (OR = 1.87; 95% CI: 1.13–3.09; *P* = 0.02). However, rates of reduction in symptoms, as assessed on the basis of the LCS scores, were similar in patients who received GBT and those randomized to the control groups (OR = 1.14; 95% CI: 0.92–1.42; *P* = 0.24).

## Discussion

This meta-analysis did not demonstrate improvement in clinical outcomes with the first-line GBT in advanced NSCLC in unselected patient population. Most patients included in these trials probably better reflects the population seen in daily clinical practice, majority of patients were male, smokers, non-Asian, and many had non-adenocarcinoma NSCLC.

Despite significantly higher ORR achieved with GBT in patients with positive EGFR mutation, that benefit did not lead to PFS or OS advantage in that group. Notably, the ORR gain was mainly attributed to the outcome of the Mok *et al*. trial that only included East Asian patients who were non- or light-smoker and had adenocarcinoma,[9] features that signals a likelihood of clinical benefit from gefitinib. The lack of survival advantage for GBT among those with EGFR mutation could be attributed to the fact that only a fraction of patients had known mutation status. Two hypotheses have been proposed as most likely to explain the negative results of GBT: (1) lack of patient selection for the target, and (2) a negative interaction between EGFR tyrosine kinase inhibitors and chemotherapy when given concurrently.[20]

In the present meta-analysis, there were insufficient data to allow analysis of PFS or OS according to other prognostic features. Clinical profiles of females, never smokers, adenocarcinoma histology, and Asian ethnicity have all been recognized as favorable subgroups that respond to gefitinib.[21–23] Higher EGFR mutation rates are also noted in these subgroups and are also related to a better response to EGFR-TKIs.[7,24] Nevertheless, the potential gender effect was not demonstrated in the INTACT-2 study.[5] Moreover, in the study of Takeda *et al*.,[16] the benefit of adding gefitinib to platinum-doublet chemotherapy was only shown for smokers, while never-smokers showed no significant benefit. The latter represents a sharp contrast to the benefit shown among never-smoker in the ISEL study.[25]

On the other hand, as compared with control interventions, GBT showed significant improvement in QOL. For patients with advanced disease, QOL and symptom relief represent important clinical end points, because a definitive cure is not achievable. More patients in the GBT than in the control had an improvement in QOL as assessed by scores on the FACT-L questionnaire and by scores on the TOI. The benefit was demonstrated in the two studies that compared gefitinib vs. chemotherapy,[9,18] or in the only study that compared gefitinib against placebo.[19] However, rates of reduction in symptoms, as assessed on the basis of the LCS scores, were similar in patients who received GBT and those randomized to control groups. Nevertheless, the involved cost and the inherent side effects, cannot be justified using gefitinib in that setting to attain some improvement in QOL. In a recent review, Neal *et al*. proposed a strategy is to move these agents to the frontline setting only for select patients.[26]

We conclude that based on the current meta-analysis, GBT cannot be recommended for the management of patients with advanced NSCLC in the first-line setting as compared with other standard interventions in unselected patient population. The significant improvement in QOL shown with GFT would be offset by the involved cost and the potential side effects known to be associated with the use of gefitinib.
